# Data of the constructivist practices in the learning environment survey from engineering undergraduates: An exploratory factor analysis

**DOI:** 10.1016/j.dib.2021.107522

**Published:** 2021-10-28

**Authors:** Chengcheng Li, Shaoan Zhang, Tiberio Garza, Yingtao Jiang

**Affiliations:** a Faculty of Foreign Languages, The Open University of China, China; b Department of Teaching and Learning, University of Nevada, Las Vegas, United States; c Department of Educational Psychology and Higher Education, University of Nevada, Las Vegas, United States; d Department of Electrical and Computer Engineering, University of Nevada, Las Vegas, United States

**Keywords:** Constructivism, Learning environment, Engineering education, First-year experience

## Abstract

This paper presents the dataset of a questionnaire on first-year engineering undergraduates’ perceptions of constructivist practices in the learning environment. The questionnaire with a 5-Likert scale was adapted from previous research. The sample consisted of 293 first-year engineering undergraduates in the southwest region of the United States. The online questionnaire was sent to participants who completed it voluntarily at the end of Fall 2019. A total of 274 of 293 participants completed the questionnaire with a response rate of 93.515%. Exploratory factor analysis was conducted to test the underlying factor structure of the questionnaire, which serves as a good reference for future research.

## Specifications Table

**Table:** 

Subject	Social sciences
Specific subject area	Education
Type of data	Tables
How data were acquired	Survey with a questionnaire (included in supplementary file)
Data format	Raw
Parameters for data collection	Participants were first-year engineering undergraduates and enrolled in a redesigned first-year experience course at a public university in the United States. Participants completed the survey voluntarily and their participation did not relate to their grade.
Description of data collection	Data were collected via an online questionnaire (Qualtrics), which was distributed through the link sent by the instructors via email at the end of the Fall 2019 semester. The questionnaire was adapted from the Constructivist Practices in the Learning Environment survey (CPLE; Tenenbaum et al., 2001). A total of 274 of 293 submitted the CPLE survey with a response rate of 93.515%.
Data source location	City/Town/Region: Southwest of the United States Country: The United States
Data accessibility	Data were included in supplementary file

## Value of the Data

• The data provides information on engineering undergraduates’ demographic information and perceptions of constructivist practices in the learning environment, which can aid survey design research and examine student responses based on demographics.
• The data also provides the area of survey item improvement and comparison to other surveys, which can aid researchers to study psychometrics in assessing engineering undergraduate in a CPLE setting.
• The data is a source for future studies on the interrelations and validity between the subscales of the CPLE survey to better understand the constructivist practices in the learning environment.
• The data is a source for subsequent studies on the comparison of CPLE setting (e.g., creating new composite variables from survey items) among engineering undergraduates to enrich the knowledge and practices of the constructivist learning environment.

## 1 Data Description

The constructivist learning environments focus on the deeper understanding through the involvement of students’ ideas [1]. Curriculum reforms across countries aim to foster students’ in-depth understanding and higher-order cognitive thinking and advocate integrating the constructivist principles into teaching and learning [2]. Several instruments have been developed to measure the learning environment in classrooms, such as the What Is Happening in This Class (WIHIC) survey [3] and the Constructivist Learning Environment Survey (CLES) [4]. However, seldom instruments were developed to evaluate the constructivist learning environment in higher education. The CPLE survey was developed by Tenenbaum et al. [5] to examine the higher-education constructivist settings. Thus, it was adapted to collect data on first-year engineering undergraduates’ perceptions of the constructivist practices in the learning environment.

The supplementary dataset provided the raw data, which was collected from the first-year engineering undergraduates in Fall 2019 and included their demographic information and perceptions of the constructivist practices in the learning environment. A questionnaire was developed and distributed to students online in a redesigned first-year experience course which included freshmen in engineering and computer science majors in a constructivist learning environment. The participants spent approximately 5–10 minutes completing the questionnaire in class. The questionnaire was voluntary and participating in this work did not have any effect on participant's final grade. A total of 274 of 293 participants completed the questionnaire with a response rate of 93.515%.

The questionnaire included two sections: demographic information and the adapted CPLE survey. The first section consisted of demographic information related to students’ age, gender, ethnicity, first-generation status, and first language. The questionnaire is provided as a supplementary file. Participants’ demographic information is shown in Table 1.

**Table 1:** Demographic information (*N* = 274).

	N	%
**Age (years)**		
<18	5	1.819
18-19	247	89.818
>19	19	6.910
**Gender**		
Male	234	85.401
Female	38	13.869
Other	1	.365
**Ethnicity**		
Hispanic/Latino	65	23.723
White/Caucasian	81	29.562
Black/African American	16	5.839
Native Hawaiian/Other Pacific Islander	10	3.650
Asian	85	31.022
American Indian or Alaska Native	1	.365
Other	14	5.109
**First generation**		
Yes	128	46.715
No	145	52.920
**English as the first language**		
Yes	209	76.277
No	64	23.358

The second section consisted of the adapted CPLE survey with 30 items. Exploratory Factor Analysis (EFA) was conducted to explore the underlying factor structure for the adapted CPLE survey. The 30 items of the CPLE survey were analyzed using Principal Component Analysis (PCA) with SPSS 26.0. Inspection of the correlation matrix showed the presence of many coefficients of .30 and above. The KMO value was .961, which exceeds the recommended value of .60 [6,7], and Bartlett's Test of Sphericity [8] was statistically significant, which supports the factorability of the correlation matrix. The number of factors was fixed to seven in SPSS since the original CPLE survey [5] included seven factors. The original F5 (Motivation toward reflections and concept investigation, Q16-Q21) of the CPLE survey (Tenebaum et al., 2001) were clustered together; however, the other items were mixed together. Thus, the original F5 (6 items including Q16-21) was kept intact and the other items (24 items including Q1-Q15 and Q22-Q30) were extracted using PCA with six fixed factors.

Based on the theoretical conception and the PCA results, new factors of the adapted CPLE survey with 30 items were proposed (see Tables 2 and 3). Table 2 shows the six new factors with their respective items and Cronbach's α, and Table 3 presents the correlation matrix of the six new factors. In Table 3, F2 was negatively and weakly related to F3 (*r* = −.043, *p* = .484) and F4 (*r* = -.014, *p* = .823), positively and weakly related to F5 (*r* = .045, *p* = .459) and F6 (*r* = .025, *p* = .685), and does not correlate with F1 (*r* = .000, *p* = .998). Thus, F2 (Conceptual conflicts and dilemmas, Q6-Q8) was deleted from the adapted CPLE survey.

**Table 2:** Factors of the adapted CPLE (30 items) (EFA results).

Factor	Item	Cronbach's α
F1 Arguments, discussion, debates	Q1, Q2, Q3	.761
F2 Conceptual conflicts and dilemmas	Q6, Q7, Q8	.618
F3 Sharing ideas with others	Q9, Q10, Q11, Q12, Q15	.842
F4 Making meaning, real-life examples	Q5, Q29, Q30	.795
F5 Motivation toward reflections and concept investigation	Q16, Q17, Q18, Q19, Q20, Q21	.917
F6 Students’ needs-based curriculum and instruction	Q4, Q13, Q14, Q22, Q23, Q24, Q25, Q26, Q27, Q28	.937

**Table 3:** Correlation matrix of the adapted CPLE (30 items).

	F1	F2	F3	F4	F5	F6
F1 Arguments, discussion, debates	1					
F2 Conceptual conflicts and dilemmas	.000	1				
F3 Sharing ideas with others	.677^⁎⁎^	−.043	1			
F4 Making meaning, real-life examples	.618^⁎⁎^	−.014	.639^⁎⁎^	1		
F5 Motivation toward reflections and concept investigation	.646^⁎⁎^	.045	.723^⁎⁎^	.667^⁎⁎^	1	
F6 Students’ needs-based curriculum and instruction	.658^⁎⁎^	.025	.770^⁎⁎^	.672^⁎⁎^	.860^⁎⁎^	1

*Note:* ** *p* < .01.

After deleting F2 (Q6-Q8), the 27 items were extracted with five fixed factors, and the results showed that Q16-Q20 (5 items from F5 Motivation toward reflections and concept investigation) were clustered together. Thus, Q16-Q20 were kept intact as F5 (Motivation toward reflections and concept investigation). Because Q21 (The course motivated me to engage in further learning of related subjects) was not clustered with F5 and has a weakly theoretical relation with the other four factors (i.e., F1-F4), Q21 was deleted from the adapted CPLE survey. Then, the remaining 21 items (Q1-Q5, Q9-Q15, and Q22-Q30) were extracted with four fixed factors and the results indicated that Q10 (The course contained a variety of learning activities) was weakly related to either component 1 or component 2, and Q15 (The course included relevant examples) was weakly related to either component 2 or component 3. Thus, Q10 and Q15 were deleted from the adapted CPLE survey. Then 19 items (Q1-Q5, Q9, Q11-Q14, and Q22-Q30) were extracted with four fixed factors. Table 4 shows the loadings of each item (Q1-Q5, Q9, Q11-Q14, and Q22-Q30).

**Table 4:** Components (varimax rotation) (Q1-Q5, Q9, Q11-Q14, and Q22-Q30, Adapted CPLE).

Component and item	Loading
*Component 1*	10 items
23. I felt pleased with what I learned in the course	.820
24. The challenging tasks in the course improved my learning	.815
4. I learned to develop cognitive tools for academic success in this course (e.g., critical thinking)	.737
27. The learning environment encouraged me to think	.705
28. The course provided meaningful examples of course concepts	.695
13. The course taught me how to arrive at appropriate answers	.692
14. The course resources effectively conveyed information that was expected to be learned	.688
26. The course helped me to pursue personal goals	.680
22. The course took into consideration my needs and concerns during class	.679
25. The course was flexible enough to accommodate my needs	.557
*Component 2*	3 items
29. The course addressed real-life events	.806
30. The course was rich in examples	.760
5. Multiple perspectives of situations were often presented in the course	.561
*Component 3*	3 items
9. The course allowed social interaction	.751
12. I was given sufficient opportunities to share my own experiences with others	.737
11. I was given sufficient opportunities to express myself	.597
*Component 4*	3 items
1. The course allowed for arguments, discussions, and debates	.797
2. The course encouraged originality of ideas	.628
3. The course allowed for constant exchange of ideas between student and instructor(s)	.561

Together with F2 (Q16-Q20) and the four factors shown in Table 4, EFA extracted five factors with 24 items of the adapted CPLE survey. Table 5 shows the five factors with their respective percentage of variance explained, Cronbach's α, mean, and standard deviation. Table 6 demonstrates the correlation matrix of the five factors of the adapted CPLE survey.

**Table 5:** Factors of the adapted CPLE (24 items) (EFA results).

Factor	EFA loading item	% of variance explained	Cronbach's α	M	SD
F1 Arguments, discussion, debates	Q1, Q2, Q3	67.857	.761	3.757	.630
F2 Sharing ideas with others	Q9, Q11, Q12	71.194	.797	3.818	.829
F3 Making meaning, real-life examples	Q5, Q29, Q30	71.157	.795	3.961	.781
F4 Motivation toward reflections and concept investigation	Q16, Q17, Q18, Q19, Q20	73.869	.911	3.664	.875
F5 Students’ needs-based curriculum and instruction	Q4, Q13, Q14, Q22, Q23, Q24, Q25, Q26, Q27, Q28	64.142	.937	3.604	.844

*Note:* Percentage of variance explained was calculated by per factor separately; M = Mean; SD = Standard deviation.

**Table 6:** Correlation metrix of the adapted CPLE (24 items).

	F1	F2	F3	F4	F5
F1 Arguments, discussion, debates	1				
F2 Sharing ideas with others	.352^⁎⁎^	1			
F3 Making meaning, real-life examples	.292^⁎⁎^	.573^⁎⁎^	1		
F4 Motivation toward reflections and concept investigation	.452^⁎⁎^	.637^⁎⁎^	.669^⁎⁎^	1	
F5 Students’ needs-based curriculum and instruction	.418^⁎⁎^	.678^⁎⁎^	.672^⁎⁎^	.848^⁎⁎^	1

*Note:* ** *p* < .01.

## 2 Experimental Design, Materials and Methods

The questionnaire was adapted from the Constructivist Practices in the Learning Environment (CPLE) survey [5] by rewording the items to be suitable for the context of engineering education. The adapted CPLE survey still kept the seven factors with 30 items: (F1) Arguments, discussions, debates; (F2) Conceptual conflicts and dilemmas; (F3) Sharing ideas with others; (F4) Materials and resources targeted towards solutions; (F5) Motivation toward reflection and concept investigation; (F6) Meeting students’ needs; and (F7) Making meaning, real-life examples. A frequency option format (1 = Never; 2 = Seldom; 3 = Sometimes; 4 = Usually; 5 = Always) was used for all items. All questionnaire questions and text from the experiment are included in this article.

The participants of this work were first-year engineering undergraduates who enrolled in a redesigned first-year experience course in a southwest region of the United States. This course allowed students of all engineering majors to be in the same class to receive lectures and conduct activities across disciplines (e.g., computer science, electrical and computer engineering, mechanical engineering, civil and environmental engineering and construction) to create a constructivist learning environment. In Fall 2019, 293 students enrolled in this course and participated in this work. The participation in this work was voluntary and had no consequences for participants’ final grade in the course. The questionnaire was distributed via an online tool (Qualtrics) and sent by the instructors via Webcampus at the end of the Fall 2019 semester. It took participants about 5–10 minutes to complete the survey. In total, 274 of 293 (93.515%) participants submitted the questionnaire. Exploratory Factor Analysis (EFA) was conducted to test the underlying factor structure of the adapted CPLE survey. Data were analyzed using SPSS 26.0.

## Ethics Statement

The work was conducted with the approval of the authors’ Social/Behavioural Institutional Review Board (IRB). The work used secondary assessment data from students enrolled in the course. There is no need to recruit the participants nor collect any additional data for this work. The authors received informed consent from all participants. Participation was voluntary and anonymous. Participants could withdraw from the work at any time. The data we submitted does not identify participants according to their responses. No identifiable information was collected by the instructors and the instructors were not able to access the data. The completion of the questionnaire had no consequences for the participants’ final grade.

## Declaration of Competing Interest

The authors declare that they have no known competing financial interests or personal relationships which have or could be perceived to have influenced the work reported in this article.

## Appendix Supplementary materials

Image, application 1

Image, application 2
