# Supplementary material for: Data of the constructivist practices in the learning environment survey from engineering undergraduates: An exploratory factor analysis
Source: Data Brief. 2021 Oct 28;39:107522. doi: 10.1016/j.dib.2021.107522 (PMC8572871; doi:10.1016/j.dib.2021.107522)
Supplement: Supplementary file 1 [file mmc1.pdf]

Constructivist Practices in the Learning Environment

Start of Block: Demographic Information

For the following questions, please select the information that you identify with/describes you the most.

Q1 What is your age?

____

Q2 What is your gender?

○ Male

○ Female

○ Other

○ Prefer not to answer

Q3 What is your race or ethnicity?

- ◯ Hispanic/Latino
- ◯ White/Caucasian
- ◯ Black/African American
- ◯ Native Hawaiian/Other Pacific Islander
- ◯ Asian
- ◯ American Indian or Alaska Native
- ◯ Other

Q4 Are you a first-generation college student?

- ◯ Yes
- ◯ No

Q5 Is English your native (first) language?

- ◯ Yes
- ◯ No

End of Block: Demographic Information

Start of Block: Constructivist Practices in the Learning Environment (CPLE)

Below is a list of statements presented that may describe the constructivist practices in the learning environment. Select the answer that most closely matches how you feel. There are no incorrect answers—we are interested in your views.

Q1 The course allowed for arguments, discussions, and debates.

- Never
- Seldom
- Sometimes
- Usually
- Always

Q2 The course encouraged originality of ideas.

- Never
- Seldom
- Sometimes
- Usually
- Always

Q3 The course allowed for constant exchange of ideas between student and instructor(s).

- ○ Never
- ○ Seldom
- ○ Sometimes
- ○ Usually
- ○ Always

Q4 I learned to develop cognitive tools for academic success in this course (e.g., critical thinking).

- ○ Never
- ○ Seldom
- ○ Sometimes
- ○ Usually
- ○ Always

Q5 Multiple perspectives of situations were often presented in the course.

- Never
- Seldom
- Sometimes
- Usually
- Always

Q6 The course caused adverse challenges to my understanding of engineering.

- Never
- Seldom
- Sometimes
- Usually
- Always

Q7 The course caused confusion in me about engineering concepts.

- Never
- Seldom
- Sometimes
- Usually
- Always

Q8 The course gave me conflicting ideas about engineering concepts.

- Never
- Seldom
- Sometimes
- Usually
- Always

Q9 The course allowed for social interaction.

- Never
- Seldom
- Sometimes
- Usually
- Always

Q10 The course contained a variety of learning activities.

- Never
- Seldom
- Sometimes
- Usually
- Always

Q11 I was given sufficient opportunities to express myself.

- Never
- Seldom
- Sometimes
- Usually
- Always

Q12 I was given sufficient opportunities to share my own experiences with others.

- Never
- Seldom
- Sometimes
- Usually
- Always

Q13 The course taught me how to arrive at appropriate answers.

- Never
- Seldom
- Sometimes
- Usually
- Always

Q14 The course resources effectively conveyed information that was expected to be learned.

- Never
- Seldom
- Sometimes
- Usually
- Always

Q15 The course included relevant examples.

- Never
- Seldom
- Sometimes
- Usually
- Always

Q16 The course motivated me to think reflectively.

- Never
- Seldom
- Sometimes
- Usually
- Always

Q17 The course encouraged me to examine multiple perspectives on issues related to engineering.

- Never
- Seldom
- Sometimes
- Usually
- Always

Q18 The ideas in the course motivated me to learn.

- ◯ Never
- ◯ Seldom
- ◯ Sometimes
- ◯ Usually
- ◯ Always

Q19 The course taught me to investigate essential concepts.

- ◯ Never
- ◯ Seldom
- ◯ Sometimes
- ◯ Usually
- ◯ Always

Q20 The course enabled me to use knowledge acquired for abstract thinking.

- Never
- Seldom
- Sometimes
- Usually
- Always

Q21 The course motivated me to engage in further learning of related subjects.

- Never
- Seldom
- Sometimes
- Usually
- Always

Q22 The course took into consideration my needs and concerns during class.

- ○ Never
- ○ Seldom
- ○ Sometimes
- ○ Usually
- ○ Always

Q23 I felt pleased with what I learned in the course.

- ○ Never
- ○ Seldom
- ○ Sometimes
- ○ Usually
- ○ Always

Q24 The challenging tasks in the course improved my learning.

- Never
- Seldom
- Sometimes
- Usually
- Always

Q25 The course was flexible enough to accommodate my needs.

- Never
- Seldom
- Sometimes
- Usually
- Always

Q26 The course helped me to pursue personal goals.

- Never
- Seldom
- Sometimes
- Usually
- Always

Q27 The learning environment encouraged me to think.

- Never
- Seldom
- Sometimes
- Usually
- Always

Q28 The course provided meaningful examples of course concepts.

- Never
- Seldom
- Sometimes
- Usually
- Always

Q29 The course addressed real-life events.

- Never
- Seldom
- Sometimes
- Usually
- Always

Q30 The course was rich in examples.

- Never
- Seldom
- Sometimes
- Usually
- Always

End of Block: Constructivist Practices in the Learning Environment (CPLE)
